# Reduction of Acquisition time using Partition of the sIgnal Decay in Spectroscopic Imaging technique (RAPID-SI)

**DOI:** 10.1371/journal.pone.0207015

**Published:** 2018-11-07

**Authors:** Sourav Bhaduri, Patricia Clement, Eric Achten, Hacene Serrai

**Affiliations:** 1 Department of Radiology and Nuclear Medicine, University of Ghent, Gent, BE; 2 Robarts Research Institute, University of Western Ontario, London, Ontario Canada; McLean Hospital, UNITED STATES

## Abstract

To overcome long acquisition times of Chemical Shift Imaging (CSI), a new Magnetic Resonance Spectroscopic Imaging (MRSI) technique called Reduction of Acquisition time by Partition of the sIgnal Decay in Spectroscopic Imaging (RAPID-SI) using blipped phase encoding gradients inserted during signal acquisition was developed. To validate the results using RAPID-SI and to demonstrate its usefulness in terms of acquisition time and data quantification; simulations, phantom and *in vivo* studies were conducted, and the results were compared to standard CSI. The method was based upon the partition of a magnetic resonance spectroscopy (MRS) signal into sequential sub-signals encoded using blipped phase encoding gradients inserted during signal acquisition at a constant time interval. The RAPID-SI technique was implemented on a clinical 3 T Siemens scanner to demonstrate its clinical utility. Acceleration of data collection was performed by inserting *R* (*R* = acceleration factor) blipped gradients along a given spatial direction during data acquisition. Compared to CSI, RAPID-SI reduced acquisition time by the acceleration factor *R*. For example, a 2D 16x16 data set acquired in about 17 min with CSI, was reduced to approximately 2 min with the RAPID-SI (*R* = 8). While the SNR of the acquired RAPID-SI signal was lower compared to CSI by approximately the factor √*R*, it can be improved after data pre-processing and reconstruction. Compared to CSI, RAPID-SI reduces acquisition time, while preserving metabolites information. Furthermore, the method is flexible and could be combined with other acceleration methods such as Parallel Imaging.

## Introduction

Chemical Shift Imaging (CSI) [1], a technique currently available as a clinical imaging tool, is used to provide tissue metabolite maps *in vivo* to help characterize neurological and metabolic disease, and improve tumour treatment [2,3].

However, CSI suffers from the time cost in obtaining these metabolite maps, and its impact on the spatial resolution and Signal to Noise Ratio (SNR) [2,3]. To overcome this problem and expand its clinical use, significant effort has been made to promote high-resolution Magnetic Resonance Spectroscopic Imaging (MRSI) techniques, using fast sequences and advanced data reconstruction methods [2–8].

These methods could be grouped into techniques which use only phase encoding gradients to obtain metabolite maps such as Multi-Spin-Echo Proton Spectroscopic Imaging technique [4,5], those using the readout gradients like echo planar spectroscopic imaging (EPSI) [6], manipulations of the shapes of the RF pulses such as wavelet encoding spectroscopic imaging technique (WE-SI) [7], or exploration of the correlation between spatial and spectral information like SPICE [8]. To further reduce acquisition time, some of the techniques cited above (e.g. WE-SI, EPSI) have been combined with Parallel Imaging (PI) [9,10]. Compressed Sensing (CS) has also been combined with blipped gradients and sophisticated post-processing algorithms to accelerate data acquisition in non-proton MRSI sequences [11].

The Reduction of Acquisition time by Partition of the sIgnal Decay in Spectroscopic Imaging (RAPID-SI) technique accelerates data acquisition by inserting a series of blipped gradients during signal acquisition without the usage of the readout gradients. In this approach, the acquired magnetic resonance spectroscopy (MRS) signal is partitioned into sequential sub-signals with short time duration, each representing one k-space data point and carrying the necessary chemical shift information. However, due to the short time duration of these sequentially acquired sub-signals, both SNR and spectral resolution are reduced. To overcome this problem, the short time encoded sub-signals are extended to full time MRS signals using a pre-processing approach described in details in the theory section.

The proposed method, implemented on a 3T Siemens whole body clinical scanner, was first demonstrated on simulated data, then validated on phantoms and *in vivo*. The method was compared to CSI to test its usefulness in terms of acquisition time, spectral resolution and data sensitivity.

This paper is organized as follows: The first section describes the technique, followed by materials and methods section describing the simulations performed along with the acquisition of *in vitro* and *in vivo* data with the scanner configurations. The results from the simulated and real MRS data are discussed and compared to those acquired using the CSI technique. Strengths and limitations of the method as compared with other methods are summarized in the discussion section, followed by a closing remark.

## Theory

### Pulse sequence

**Fig 1:**
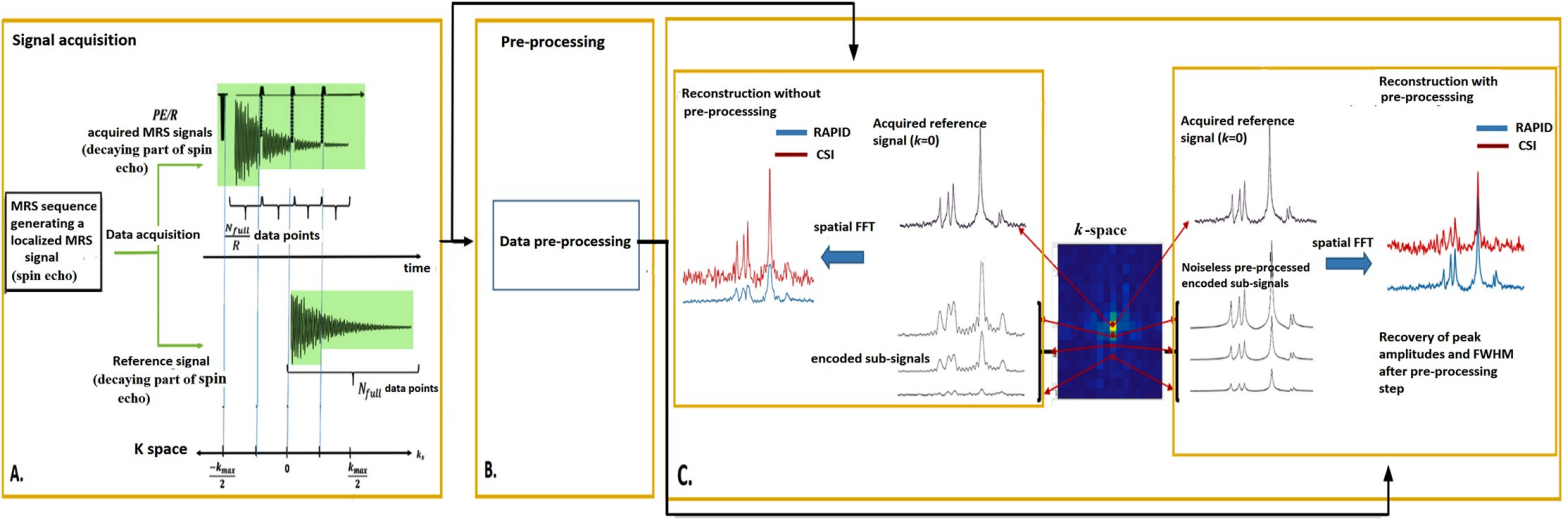
Method steps of the RAPID-SI technique on simulated MRSI data. (A) data acquisition, (B) pre-processing, (C) signal reconstruction with an example showing the reconstruction of spectra with and without the pre-processing approach. The RAPID-SI short time sub-signals without pre-processing are zero-filled to full time length when compared to the CSI.

The RAPID-SI technique acquires a predetermined number of localized spin echo MRS signals (*AS*). The decaying part of each acquired *AS* signal is partitioned into *R* successively encoded sub-signals to fill *R* subsequent k-space data points. The encoding is performed by insertion of *R* equally spaced blipped phase encoding gradients during data acquisition as shown in Fig 1. The k-space data collection accelerated by the factor *R* is given by:

$$s(k_{(l*R)+j},t_{j})=\int \bar{s}({x,t}_{j}).e^{\left(i.2.\pi .k_{(l*R)+j}.x\right)}.dx$$ (1)

where the k-space variable *k* $(k=\frac{\gamma }{2.\pi }\int_{0}^{\tau_{G}}G_{x}(t).dt$, with *τ*_*G*_ being the gradient blip duration) runs from $-\frac{\frac{PE}{2}}{FOV}$ to $\frac{(\frac{PE}{2})-1}{FOV}$ (*PE* is the total number of phase encoding steps, FOV is the field of view), and $\bar{s}({x,t}_{j})$ is the signal acquired at voxel position *x* in the MRSI grid during the time window *t*_*j*_ (*j* runs from 1 to *R*), having constant duration set to the number of data points *N*_*sub*_ = *N*_*full*_/*R* (*N*_*full*_ is the total number of points of the MRS signal). The time variable *t*_*j*_ runs from 1+(*j*−1).*N*_*sub*_ to *j*.*N*_*sub*_. The variable *l* runs from 0 to (*AS*−1), and *AS* is set to *PE*/*R*. The signal $\bar{s}({x,t}_{j})$ is given by:

$$\bar{s}({x,t}_{j})=\sum_{m=1}^{M}A_{m_{x}}.e^{-t_{j}(d_{m}+2\pi if_{m})}+\varepsilon (t_{j})$$ (2)

The parameter *M* is the number of the spectral peaks, assumed to be Lorentzian. $A_{m_{x}}$ denotes the amplitude of metabolite *m* at voxel position *x* in the MRSI grid, whereas *f*_*m*_, and *d*_*m*_, are the corresponding frequency and damping factor, respectively. The term *ε*(*t*) describes noise. Inserting Eq 2 into Eq 1, we obtain:

$$\begin{array}{l} s\left(k_{\left(l*R\right)+j},t_{j}\right)=\int \sum_{m=1}^{M}A_{m_{x}}.e^{-t_{j}\left(d_{m}+2\pi if_{m}\right)}.e^{\left(i.2.\pi .k_{\left(l*R\right)+j}.x\right)}.dx+\varepsilon \left(t_{j}\right)\\ \;=\sum_{m=1}^{M}A_{m_{k_{\left(l*R\right)+j}}}.\psi_{sub}\left(m,t_{j}\right)+\varepsilon \left(t_{j}\right) \end{array}$$ (3)

where ${A_{m_{k_{(l*R)+j}}}}_{}=\int {A_{m_{x}}}^{}.e^{i.2\pi k_{(l*R)+j}x}\;dx$, is the phase modulated amplitude, dependent on *k*, and therefore specific to each *s*(*k*_(l*R)+*j*_,*t*_*j*_), and the constant part, $\psi_{sub}({m,t}_{j})=e^{-t_{j}(d_{m}+2\pi if_{m})}$, carrying the parameters *d*_*m*_, and *f*_*m*_ of the spectral component *m* during the time window *t*_*j*_.

The proposed method is comparable to CSI when the number of inserted blips is one per acquired signal (*R =* 1). It reaches, however, its full speed when all the necessary blips are played within a single acquisition (*R = PE* and *AS* = 1). Therefore, the reduction in acquisition time is proportional to *R*.

The Signal to Noise Ratio per voxel of the CSI in one dimension is given by [2]:

$${SNR}_{CSI}=\frac{A.e^{-\tau_{G}/T_{2}^{*}}{.T}_{2}^{*}.\Delta f.(1-e^{-N_{full}/T_{2}^{*}})}{a.\sqrt{N_{x}}.\sqrt{N_{full}\Delta f}}$$ (4)

where *A* denotes the peak amplitude of a metabolite. *N*_*x*_ is the number of voxels along *x* direction, Δ*f* is the bandwidth of the MRS spectrum, and *a* being a constant term contributing to the noise standard deviation [2]. The term $\left(1-e^{-N_{full}/T_{2}^{*}}\right)$ is related to the duration of the acquisition window. Given that acceleration is performed in the one given direction (e.g. *x*), one can deduce the SNR per voxel of the 1D RAPID-SI method accordingly:

$$SNR_{RAPID}=\frac{A.e^{-\tau_{G}/T_{2}^{*}}.\left(1+e^{-N_{full}/R.T_{2}^{*}}+e^{-2.N_{full}/R.T_{2}^{*}}+\cdots +e^{-\left(R-1\right)N_{full}/R.T_{2}^{*}}\right).T_{2}^{*}.\Delta f.\left(1-e^{-N_{full}/R.T_{2}^{*}}\right)}{a.R.\sqrt{N_{x}}.\sqrt{N_{sub}\Delta f}}$$ (5)

which can be rewritten as:

$$SNR_{RAPID}=\frac{Ae^{-\tau_{G}/T_{2}^{*}}.T_{2}^{*}.\Delta f.\left(1-e^{-N_{full}/T_{2}^{*}}\right)}{a.R.\sqrt{N_{x}}.\sqrt{N_{full}\Delta f/R}}$$ (6)

Combining Eq 4 and Eq 6, we obtain:

$${SNR}_{RAPID}=\frac{{SNR}_{CSI}}{\sqrt{R}}$$ (7)

The sensitivity of RAPID-SI in terms of SNR and acquisition time, given by $\Psi =\frac{SNR}{\sqrt{T_{tot}}}$ [2] with *T*_*tot*_ being the total acquisition time, is comparable to the CSI (Ψ_*RAPID*_ = Ψ_*CSI*_). One can increase the SNR in RAPID-SI by shortening the number of points *N*_*full*_, which could be set to the shortest *T*_2_ value of the collected metabolite signals (e.g. *N*_*full*_ ≅ *T*_2,*shortest*_). However, reducing *N*_*full*_, lowers the acceleration factor *R* since a minimum data length of *N*_*sub*_ is required to allow for an accurate estimation of the phase modulated amplitudes $A_{m_{k}}$. Therefore, to allow for higher acceleration and minimise the SNR loss, *N*_*full*_ is generally set equal to the number of data points corresponding to the averaged *T*_2_ value of the metabolite signals (*T*_2,*avg*_). At the acquisition level, the data has low SNR and low spectral resolution, and sub-signal amplitudes modulated by the decaying effect due to the sequential acquisition of these sub-signals. This sequential data acquisition leads to inaccurate data analysis. To overcome this problem, the sequentially acquired short time encoded sub-signals are extended to full time MRS signals using the pre-processing approach as described below.

### HLSVD-PRO based pre-processing

To recover the missing information due to the short time duration of the acquired sub-signals, the latter have to be extrapolated to full time length. We have chosen to use the HLSVD-PRO method to achieve this task [12].

To estimate *d*_*m*_, and *f*_*m*_ in *ψ*_*sub*_(*m*,*t*_*j*_) in Eq 3, a reference signal *AS*_0_ with full time length (*N*_*full*_) is acquired at the centre of k-space (*k* = 0) and fitted, using HLSVD-PRO, to the corresponding signal model given by:

$$s(k=0,t=N_{full})=\sum_{m=1}^{M}\begin{array}{c} A_{m_{k=0}}\;\psi_{full}(m,t)=\psi_{full}(m,t).(\begin{array}{c} A_{1_{k=0}}\\ \vdots \\ :\\ A_{M_{k=0}} \end{array}) \end{array}$$ (8)

where the term *ψ*_*full*_(*m*,*t*) is described by an (*N*_*full*_ *X M*) matrix as follows:

$$\psi_{full}(m,t)=\left(\begin{array}{ccc} e^{-t_{1}(d_{1}+2\pi if_{1})} & \cdots & e^{-t_{1}(d_{M}+2\pi if_{M})}\\ \vdots & \ddots & \vdots \\ e^{-t_{N_{full}}(d_{1}+2\pi if_{1})} & \cdots & e^{-t_{N_{full}}(d_{M}+2\pi if_{M})} \end{array}\right)$$ (9)

The generated matrix above in Eq 9, is then time sub-divided into sequentially reduced size (*N*_*sub*_ *X M*) sub-basis functions *ψ*_*sub*_(*m*,*t*_*j*_) according to the time occurrence *t*_*j*_ as follows:

$$\psi_{sub}({m,t}_{j})=\left(\begin{array}{ccc} e^{-t_{\left(1+(j-1)*N_{sub}\right)}(d_{1}+2\pi if_{1})} & \cdots & e^{-t_{\left(1+(j-1)*N_{sub}\right)}(d_{M}+2\pi if_{M})}\\ \vdots & \ddots & \vdots \\ e^{-t_{\left(j*N_{sub}\right)}(d_{1}+2\pi if_{1})} & \cdots & e^{-t_{\left(j*N_{sub}\right)}(d_{M}+2\pi if_{M})} \end{array}\right)$$ (10)

Inserting Eq 10 into Eq 3, a noiseless model for the acquired *s*(*k*_(l*R)+*j*_,*t*_*j*_) is given by:

$$\begin{array}{c} s(k_{(l*R)+j},t_{j})=\psi_{sub}({m,t}_{j}).\left(\begin{array}{c} A_{1_{k_{(l*R)+j}}}\\ \vdots \\ :\\ A_{M_{k_{(l*R)+j}}} \end{array}\right)\\ =\left(\begin{array}{ccc} e^{-t_{\left(1+(j-1)*N_{sub}\right)}(d_{1}+2\pi if_{1})} & \cdots & e^{-t_{\left(1+(j-1)*N_{sub}\right)}(d_{M}+2\pi if_{M})}\\ \vdots & \ddots & \vdots \\ e^{-t_{\left(j*N_{sub}\right)}(d_{1}+2\pi if_{1})} & \cdots & e^{-t_{\left(j*N_{sub}\right)}(d_{M}+2\pi if_{M})} \end{array}\right).\left(\begin{array}{c} A_{1_{k_{(l*R)+j}}}\\ \vdots \\ :\\ A_{M_{k_{(l*R)+j}}} \end{array}\right) \end{array}$$ (11)

**Fig 2:**
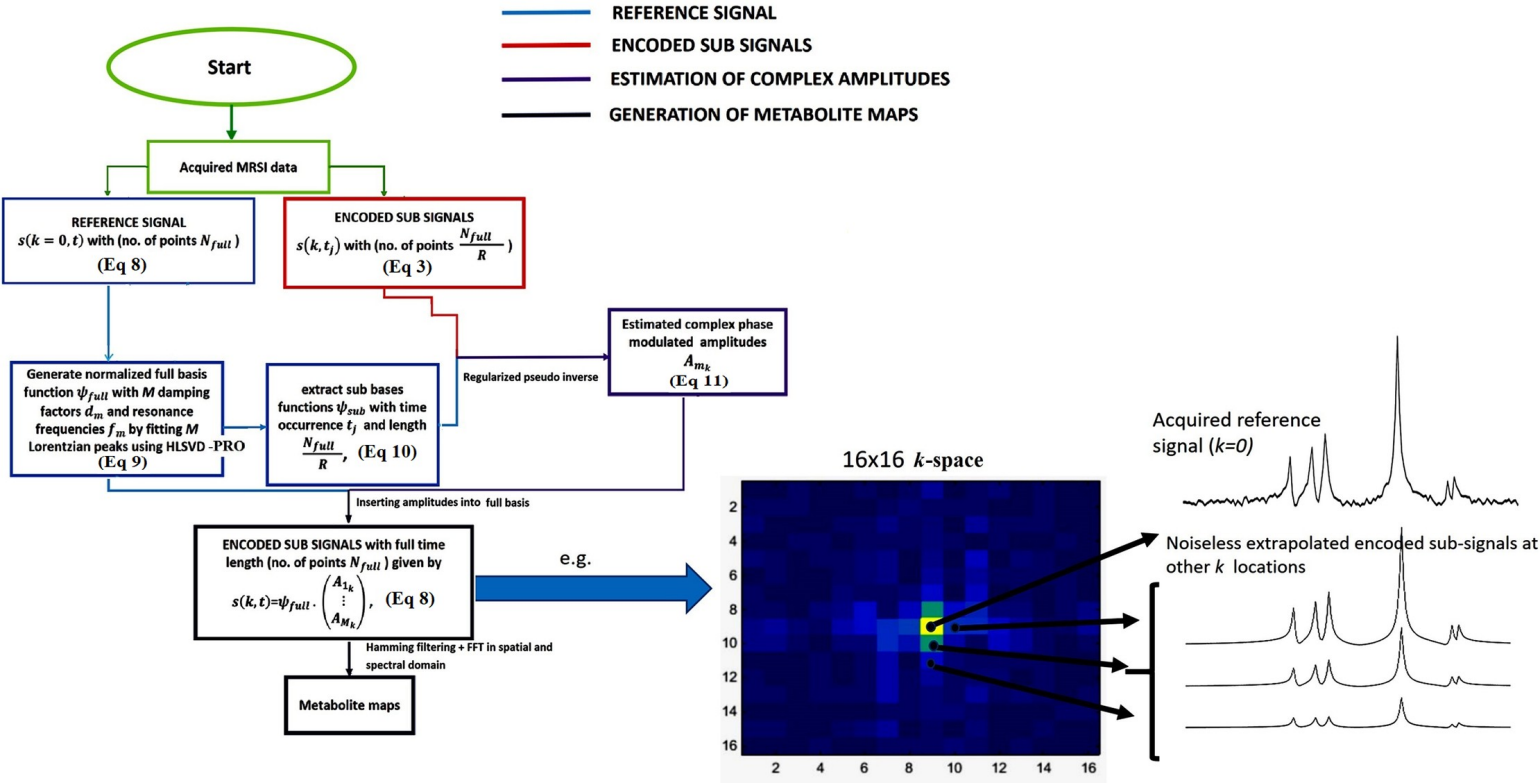
Flow diagram of the proposed RAPID-SI technique from data acquisition/partition, processing to signal reconstruction.

Then, the complex amplitudes $A_{m_{k}}=\left(\begin{array}{c} A_{1_{k_{(l*R)+j}}}\\ \vdots \\ :\\ A_{M_{k_{(l*R)+j}}} \end{array}\right)$ in Eq 11 are estimated from the corresponding acquired *s*(*k*_(l*R)+*j*_,*t*_*j*_) using a pseudo-inverse matrix operation [13]. The estimated amplitudes are used to substitute the amplitude vector $\left(\begin{array}{c} A_{1_{k=0}}\\ \vdots \\ :\\ A_{M_{k=0}} \end{array}\right)$ in Eq 8 to construct the corresponding full time noiseless *s*(*k*_(l*R)+*j*_,*t* = *N*_*full*_) signal. Fourier Transform is then performed in both spatial and temporal domain on the *PE* reconstructed $s\left(k_{-\frac{\frac{PE}{2}}{FOV},\ldots \ldots .,\frac{(\frac{PE}{2})-1}{FOV}},t=N_{full}\right)$ k-space data, including the reference signal *AS*_0_ (*k* = 0,*t* = *N*_*full*_), to obtain the metabolite maps. A schematic representation of the method is shown in Fig 1 and the algorithm used to obtain metabolite maps from the acquired data is displayed in Fig 2.

## Materials and methods

### Simulation

**Fig 3:**
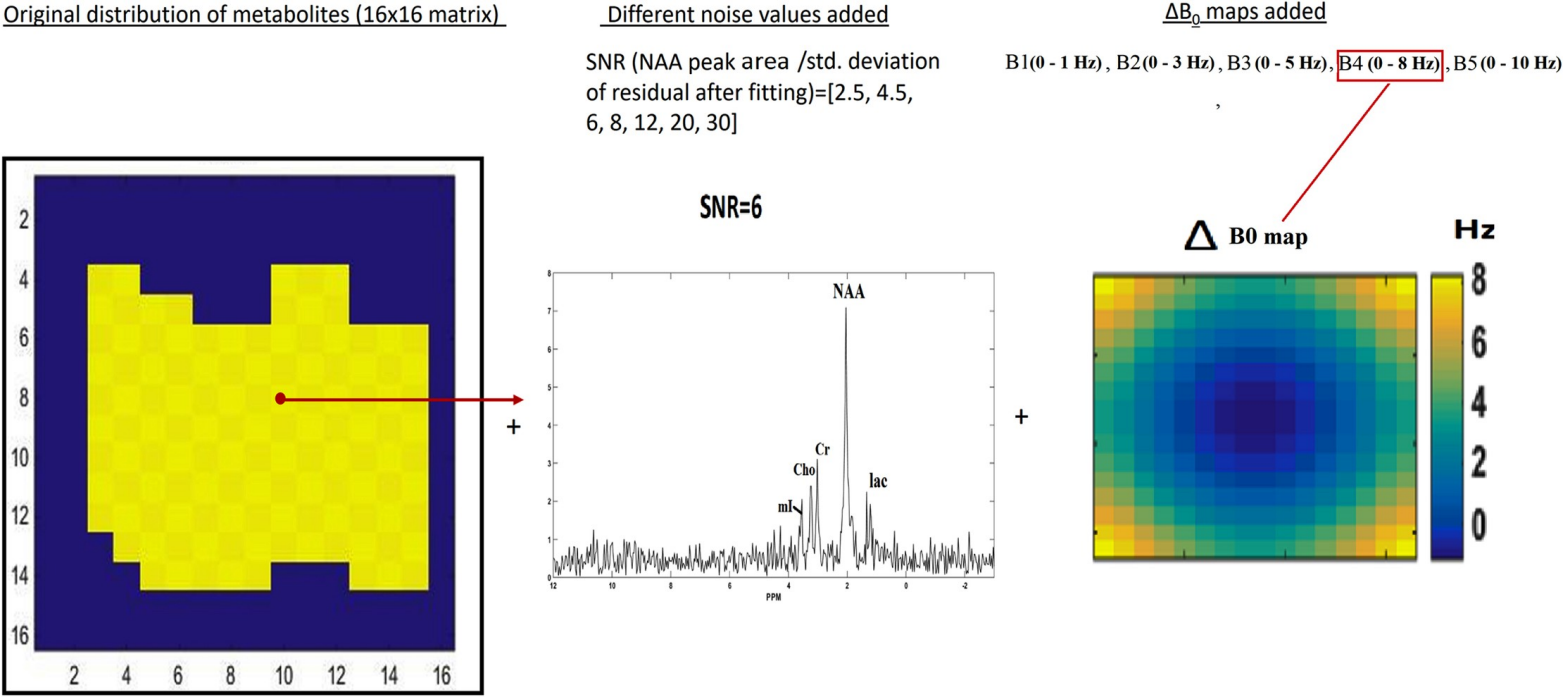
Simulated metabolite maps, with added variable noise and ΔB_0_ maps (example shown with SNR = 6 and ΔB_0_ map B4).

A two dimensional 16x16 MRSI data shown in Fig 3, was simulated with uniform distribution of brain metabolites like Choline (Cho), Creatine (Cr), N-acetylaspartate (NAA), Myo-Inositol (mI) and lactate (lac) using Lorentzian line shape with *T*_2_ (inversely proportional to damping factor (*d*_*m*_)), and resonance frequencies (*f*_*m*_) obtained from literature [14–18], according to Eq 2 using four singlet peaks for Cho, Cr, NAA, mI and a doublet for lactate. The amplitudes of Cho, Cr, NAA, mI and lactate resonances were set to 0.9, 1.06, 2.23, 0.64, 0.49, respectively. The following acquisition parameters were used: TE = 35 ms, TR = 2000 ms, BW = 2000 Hz, *N*_*full*_ = 512. A white Gaussian noise was added to the data and varied between different SNR values from 2.5, 4.5, 6, 8, 12, 20, to 30. The SNR is defined as:

$$SNR=\frac{NAA\;peak\;area}{standard\;deviation\;of\;noise}$$ (12)

The B_0_ field inhomogeneity, replicating *in vivo* conditions in the human brain was also incorporated into the data [19]. Five ΔB_0_ spatial maps (B1 –B5) were generated by varying the resonance frequencies *f*_*m*_ and linewidth *d*_*m*_ of the peaks in Eq 2, across all the voxels in the ROI by a factor ranging from 0 to 1 Hz in B1, 0 to 3 Hz in B2, 0 to 5 Hz in B3, 0 to 8 Hz in B4, 0 to 10 Hz in B5 (Fig 3). The FOV dimensions were set to AP = 100 mm and RL = 100 mm. The acceleration factor *R* was varied from 2, 4, 8, to 16.

### Sequence and hardware

The RAPID-SI sequence was accelerated in the anterior-posterior (AP) direction by inserting gradient blips during data acquisition in a PRESS sequence [20]. Regular phase encoding was applied in the Right-Left (RL) and Head-Foot (HF) directions. Water and lipid suppression were performed using CHESS and outer-volume saturation (OVS) techniques, respectively [21,22]. The sequence was implemented on a 3T Siemens whole body scanner, with a gradient strength of 45 mT/m and a nominal slew rate of 200 mT/m/s. A 32-channel receive head coil was used for data acquisition. The reference signal *AS*_0_ was acquired first from the PRESS Region of Interest (ROI) prior to encoding by turning off all the encoding gradients (*k* = 0,*N*_*full*_ = 512).

### Phantom scans

*In vitro* water suppressed and unsuppressed 2D RAPID-SI and CSI data were acquired from a cylindrical water solution phantom containing tubes filled with brain metabolite solutions (NAA, lac, Cho and Cr with known concentrations of 10.5 mM, 25 mM, 9.8 mM and 4.5 mM, respectively). Tube 1 contains Cr only, tube 2 contains NAA and lac, tube 3 contains lac and Cho and tube 4 containing lac and mI. The following acquisition parameters were used: TE = 35 ms, TR = 2000 ms, BW = 2000 Hz, *N*_*full*_ = 512, *N*_*avg*_ = 2, and *R* = 4, 8, 16. The FOV dimensions were AP = 100 mm, HF = 10 mm and RL = 100 mm for 2D acquisitions, with spatial resolution set to 16x16.

Localizer gradient echo images, with 40° flip angle, TR/TE of 20/5 ms, 10 mm thick, 256 x 128 matrix, were acquired prior to spectroscopy for the positioning of the PRESS ROI volume. Shimming was performed using Siemens software FASTMAP [23].

The water line was calculated from the unsuppressed water data and used as an internal reference to estimate the absolute concentrations of the metabolites. The absolute concentrations of the metabolites were estimated using the following equation:

$$C_{metabolite}=C_{water}.\left(\frac{2}{np}\right).\left(\frac{A_{metabolite}}{A_{water}}\right).\frac{exp(\frac{TE}{{T2}_{metabolite}})}{exp(\frac{TE}{{T2}_{water}})}.\frac{\left\{1-exp(\frac{-TR}{{T1}_{water}})\right\}}{\left\{1-exp(\frac{-TR}{{T1}_{metabolite}})\right\}}$$ (13)

and compared to the CSI. The variable *C*_*metabolite*_ is the metabolite concentration (mM), *C*_*water*_ is the water concentration used as internal reference (110 M) [24], *A*_*metabolite*_ is the area of the metabolite peak, *A*_*water*_ is the area of the water peak calculated from the unsuppressed water data, *np* is the number of protons in a given metabolite, and *T*1_*metabolite*_, *T*2_*metabolite*_, *T*1_*water*_, *T*2_*water*_ are the relaxation times of metabolites and water respectively [14–18].

### Volunteer scans

Eight healthy volunteers, aged from 20 to 30 years, were scanned using the RAPID-SI sequence. Written informed consent were obtained from each participant prior to scanning. This validation study was approved by the Ethical Committee of the Ghent University Hospital. A 2D 8x8 and 16x16 brain RAPID-SI and CSI datasets were acquired using the following parameters: TE = 35 ms, TR = 2000 ms, BW = 2000 Hz, *N*_*full*_ = 512, *N*_*avg*_ = 2, and *R* = 4, 8. The FOV dimensions were AP = 100 mm, HF = 10 mm and RL = 100 mm for 2D acquisitions. Additional 3D (16x16x4) RAPID-SI and CSI dataset were acquired from two other volunteers using the same acquisition parameters as the 2D dataset, except for TR = 1800 ms, and *N*_*avg*_ = 1. The resolution in the slice direction was reduced to 4 due to the long acquisition time required by CSI.

High-resolution T1 weighted gradient echo MR images (70° flip angle, TR/TE 255/2.96 ms, 30 slices, 3.0 mm thick, 256 x 192 matrix) from the entire head are acquired for PRESS volume positioning. Eight outer-volume saturation bands, placed around the PRESS ROI box, were used for lipid suppression and CHESS water suppression was performed. Shimming was performed using the automated software package FASTMAP [23].

### Data processing

The acquired MRSI data was processed offline using an in-house program using Matlab (MathWorks, USA). The pre-processing software with a graphical user interface for data analysis is made available (see GitHub link in data availability statement). The reference signal, *AS*_0_ (*k* = 0,*N*_*full*_ = 512), was processed first to estimate the signal parameters *d*_*m*_, and *f*_*m*_ using HLSVD-PRO. The model order *M* for HLSVD-PRO was set to 10 to include the major brain metabolites. The estimated parameters were then inserted into Eq 10 to generate the sub-bases *ψ*_*sub*_(*m*,*t*_*j*_), which were used in Eq 11, to calculate the complex amplitudes $A_{m_{k}}$ from the collected *s*(*k*_(l*R)+*j*_,*t*_*j*_) data. The calculated $A_{m_{k}}$ values were used in Eq 8 to produce the noiseless $s\;\left(k_{-\frac{\frac{PE}{2}}{FOV},\ldots \ldots .,\frac{(\frac{PE}{2})-1}{FOV}},t=N_{full}\right)$ signals. A Hamming filter was applied on the constructed $s\;\left(k_{-\frac{\frac{PE}{2}}{FOV},\ldots \ldots .,\frac{(\frac{PE}{2})-1}{FOV}},t=N_{full}\right)$ data along with *AS*_0_, prior to Fourier transform, to obtain localized spectra/metabolite maps. The sum of squares approach for coil channel combination was applied after data reconstruction. Water suppression using HLSVD-PRO was optimally achieved with model order of 7.

To accurately estimate signal parameters using HLSVD-PRO, the duration of the acquisition window was set to the average *T*_2_ values of the main brain metabolites (NAA, Cr, Cho, mI and lac) obtained at 3 Tesla, found to be approximately 250 ms [14–18] and the receiver bandwidth set to 2000 Hz, the total number of points *N*_*full*_ was set to 512 data points.

As shown on the method flow chart (Figs 1 and 2), the reference signal *AS*_0_ is always used with the noiseless pre-processed *s*(*k* (*k* ≠ 0),*t* = *N*_*full*_) signals for data reconstruction. This induces noise to be present in the output localized spectra after Fourier transform.

The fitting of metabolite peaks, was achieved using HLSVD-PRO with model order of 10. All the metabolite peaks, Cho, Cr, NAA, mI and lac, in the frequency range 1 to 4 PPM were fitted. Cramér–Rao Lower Bound (CRLB) criteria for NAA, Cr and Cho peaks was used to evaluate the quality of fitting. The CRLB/amplitude < 20% was selected as a criterion to discriminate well fitted metabolites from poorly fitted ones.

### Statistical analysis

A Wilcoxon signed-rank test [25] using SPSS Statistics 25, was applied to check on the difference between the median of NAA/Cr and Cho/Cr ratios of CSI and RAPID-SI techniques. A p-value of 0.05 below which the study was considered statistically significant. All analyses were conducted using SPSS Statistics 25.

## Results

### Simulation results

**Fig 4:**
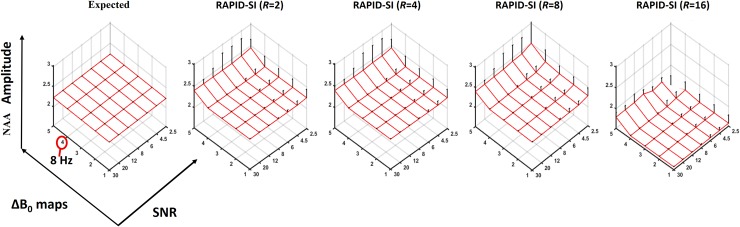
Simulation results. Expected values of mean (red mesh plot) and standard deviation (black bars on the top of mesh plot) of the amplitude of NAA peaks across all the voxels from simulated data (for SNR values [2.5, 4.5, 6, 8, 12, 20, 30] and different ΔB_0_ maps) (left) and reconstruction results with RAPID-SI (right) with different acceleration factors R = [2, 4, 8, 16] after pre-processing. In the plot, along the ΔB_0_ maps axis the numbers 1 to 5 represent ΔB_0_ maps B1 –B5, the red circle on 4 indicates the map B4 with inhomogeneity value of 8 Hz.

**Fig 5:**
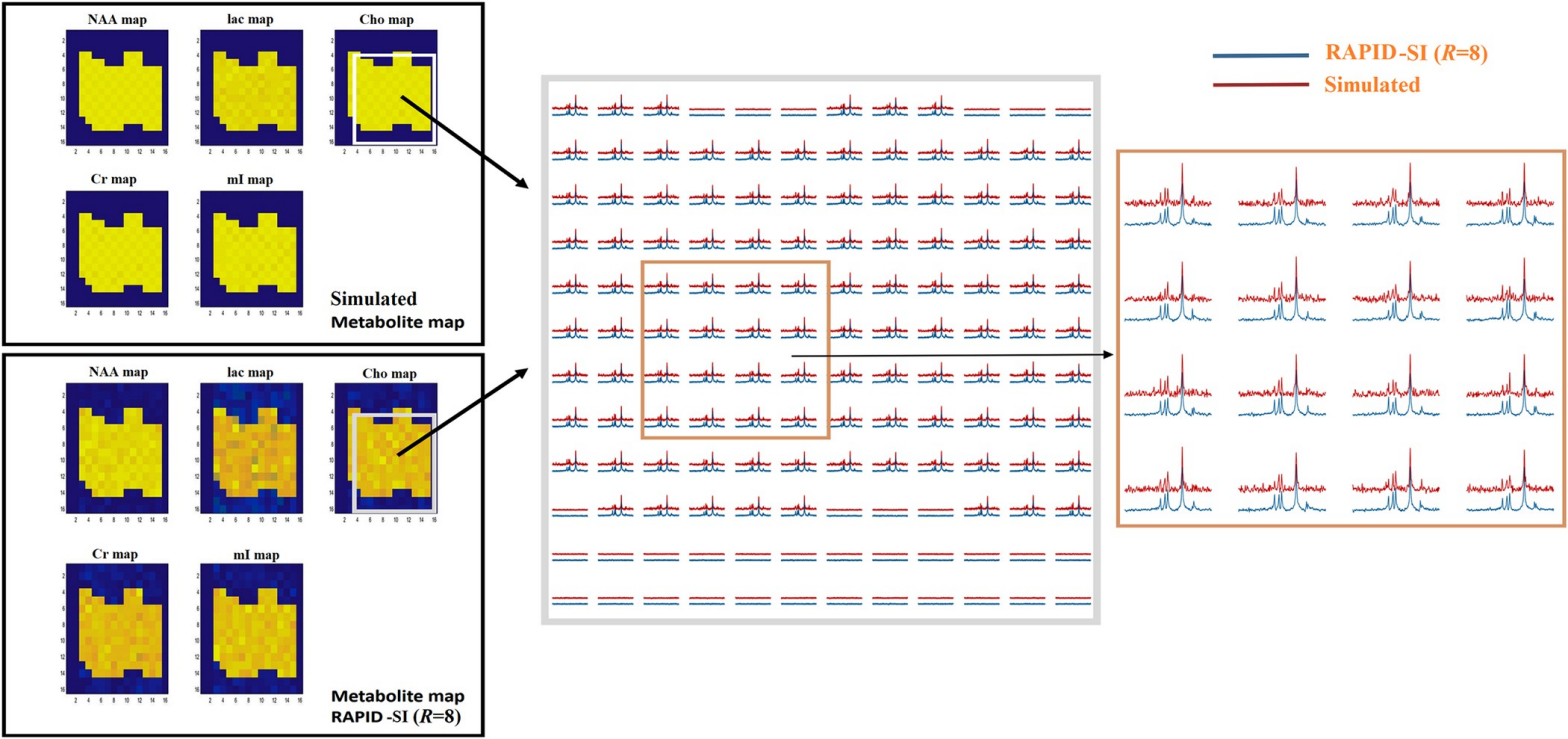
Reconstruction results of simulated data. 2D simulated (with simulation parameters of SNR = 6 and ΔB_0_ map B2) (top left) and reconstructed RAPID-SI (*R* = 8) (bottom left) metabolite maps after pre-processing. Spectra from the zoomed 12x12 matrix (white box) and the central 4x4 matrix (brown box) are displayed.

With *N*_*full*_ set to 512, the highest acceptable *R* value was 8 with an SNR of 4.5 (Fig 4). Higher acceleration factors cause quantification errors on the estimation of metabolite peaks regardless of the SNR values and/or field inhomogeneity. This was mainly due to the very short duration of the encoded sub-signals, (e.g: 32 data points, with *N*_*full*_ = 512, and *R* = 16). The lowest acceptable SNR, was established by the standard deviation of the estimated metabolite values, tolerated to deviate by about 20% from the expected ones (Fig 4). The effect of field inhomogeneity was insignificant on the accuracy of the data quantification when the level of field inhomogeneity (e.g: difference in full width at half maximum (FWHM) of the metabolite peaks from one voxel to another) is around 8 Hz (ΔB_0_ map B4). However, with a level of inhomogeneity above 8 Hz (ΔB_0_ map B5), significant quantification errors on estimated data were noticed (Fig 4). Therefore, to obtain accurate results with *N*_*full*_ = 512, and *R* set to 8, the SNR should be ≥ 4.5 and the level of field inhomogeneity ≤ 8 Hz. These values were compared to those obtained from *in vivo* MRSI datasets collected from healthy volunteers using the same acquisition parameters and with several spatial resolutions and dimensions. The SNR *in vivo* was found to be 8.3 in the centre of the brain and decreases to about 6 ~ 7 at the edges of the region of interest (ROI), producing a peak broadening of about 3 Hz for the NAA, Cho and Cr resonances (S1 Fig). Therefore, the limits of the proposed method in term of SNR and field inhomogeneity were within those encountered *in vivo*, making RAPID-SI feasible in clinical settings. As shown in Fig 5, comparable metabolite maps for NAA, lac, Cho, Cr and mI were obtained from RAPID-SI (*R* = 8) with respect to the simulated data for SNR value of 6 and ΔB_0_ map B2.

### Phantom results

**Fig 6:**
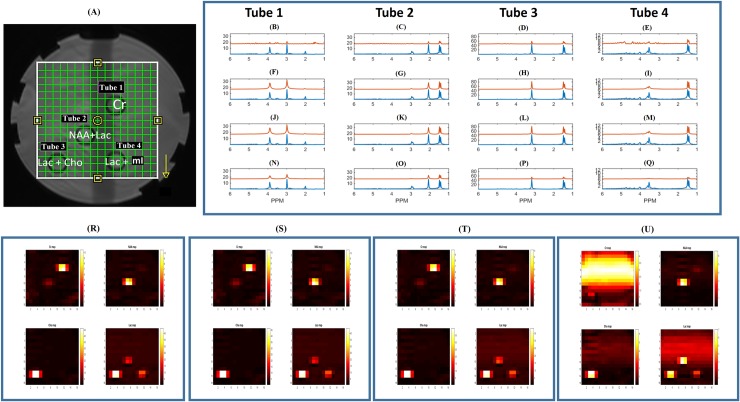
*In vitro* 2D phantom data results. (A) Phantom setup containing 4 tubes with different metabolite distributions. (B—E) showing the reconstructed metabolite peaks in Tubes 1–4 from raw CSI data (blue) and the raw RAPID-SI data (red), zero-filled to full time length of 512 points and with acceleration factor *R* = 8, (F—I) showing the reconstructed metabolite peaks in Tubes 1–4 from raw CSI data (blue) and pre-processed RAPID-SI data (red) with *R* = 4, (J—M) showing the reconstructed metabolite peaks in Tubes 1–4 from raw CSI data (blue) and the pre-processed RAPID-SI data (red) with *R* = 8, and (N–Q) showing the reconstructed metabolite peaks in Tubes 1–4 from raw CSI data (blue) and the pre-processed RAPID-SI data (red) with *R* = 16. Metabolite maps with color-bar representing the metabolite peak amplitudes of Cr, NAA, Cho and Lac from the white ROI using (R) raw CSI data, (S) pre-processed RAPID-SI (*R* = 4), (T) pre-processed RAPID-SI (*R* = 8), (U) pre-processed RAPID-SI (*R* = 16).

Fig 6A shows the 4 tubes within the phantom with different metabolite distributions. In Fig 6R–6U and 2D 16x16 RAPID-SI and CSI *in vitro* results, are displayed, with comparable metabolite distributions for NAA, lac and Cho, Cr across all the tubes up to acceleration factor of 8, after pre-processing (Fig 6T). Furthermore, RAPID-SI provided accurate quantification results (Table 1) up to acceleration factor of 8, after pre-processing. However, higher than 8, the quantification accuracy degrades as seen from the spectra in Fig 6N–6Q and in the metabolite map in Fig 6U. It can also be seen in Fig 6B–6E (comparing reconstructed metabolite peaks from raw CSI data (blue) and the raw RAPID-SI data (red), zero-filled to full time length of 512 points) that the raw data acquired using RAPID-SI results in inaccurate reconstruction of the peaks due to the low number of points of the sub-signals, which is recovered after the pre-processing step as seen in Fig 6F–6I (with *R* = 4) and in Fig 6J–6M (with *R* = 8) respectively. The concentrations of the metabolites were calculated according to Eq 13. There was a significant reduction in acquisition time (17 min with CSI and about 2 min with the RAPID-SI (*R* = 8)).

**Table 1:** Mean value of expected and estimated metabolite concentration values from CSI and RAPID-SI for different acceleration factors.

	NAA (mM)	Lac (mM)	Cho (mM)	Cr (mM)
**Expected**	10.5	25	9.8	4.5
**CSI**	10.3	25.1	9.7	4.6
**RAPID-SI (*R* = 4)**	10.3	25.2	9.9	4.6
**RAPID-SI (*R* = 8)**	10.2	24.7	9.4	4.3
**RAPID-SI (*R* = 16)**	5.1	13.2	3.3	1.5

### *In vivo* results

The overall SNR as defined in Eq 12 was lower in RAPID-SI than CSI by approximately the factor √R at the acquisition level. The SNR mean ± standard deviation (SD) in CSI across the volunteers was 6.98 ± 0.2, and reduced to 2.32 ± 0.15 in RAPID-SI with (*R* = 8). The calculated SNR using both the methods for all the volunteers can be found in S1 Appendix.

**Fig 7:**
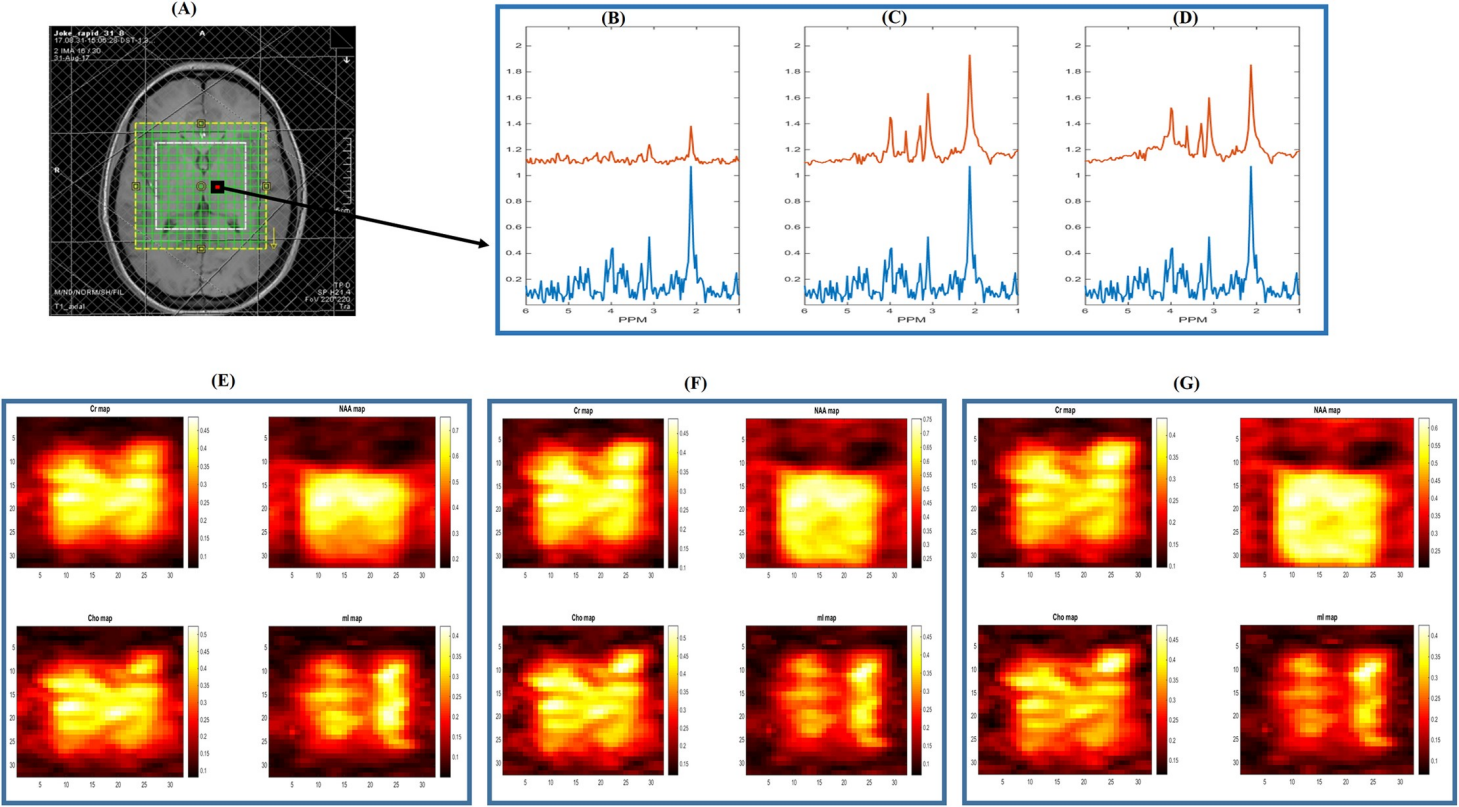
2D 16x16 *in vivo* data results. (A) 2D 16x16 *in vivo* data with the slice FOV (yellow box) and volume of interest (white box), (B) displaying the reconstructed metabolite peaks in a voxel (indicated in red in (A)) from raw CSI data (blue) and the raw RAPID-SI data (red), zero-filled to full time length of 512 points with *R* = 8, (C) showing the reconstructed metabolite peaks in the same voxel from the raw CSI data (blue) and the pre-processed RAPID-SI data (red) with *R* = 4, (D) showing the reconstructed metabolite peaks in the same voxel from the raw CSI data (blue) and pre-processed RAPID-SI data (red) with *R* = 8. Metabolite maps (interpolated to a resolution of 32 x 32) with color-bar representing peak amplitudes of Cr, NAA, Cho and mI from the FOV using (E) raw CSI data, (F) RAPID-SI (*R* = 4), (G) RAPID-SI (*R* = 8).

Similar results (Fig 7E–7G) in terms of metabolite maps *in vivo* of Cr, NAA, Cho and mI, were obtained, demonstrating that RAPID-SI was able to preserve the spatial distribution of the metabolites while reducing acquisition time. It can also be seen when comparing reconstructed metabolite peaks from raw CSI data (blue) and the raw RAPID-SI data (red), zero-filled to full time length of 512 points (Fig 7B) that the raw data acquired using RAPID-SI results in inaccurate peaks reconstruction due to low number of data points of the sub-signals, which is recovered by the pre-processing step as displayed in Fig 7C and 7D with RAPID-SI (*R* = 4) and (*R* = 8) respectively.

**Fig 8:**
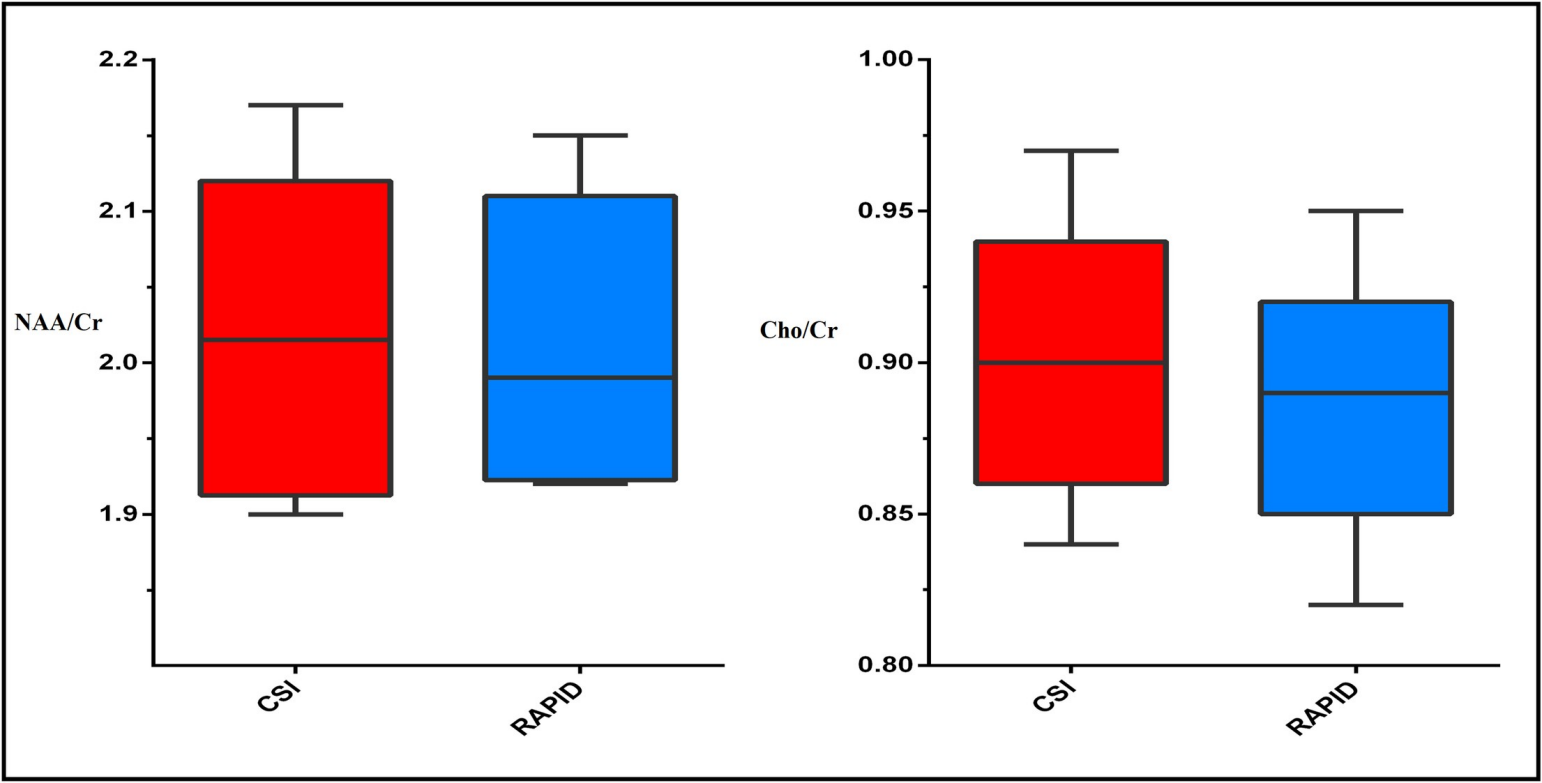
Box plot showing the statistical comparison of the mean *in vivo* NAA/Cr (left) and Cho/Cr (right) ratios across all the volunteers for CSI and RAPID-SI (*R* = 8).

The pre-processed RAPID-SI data were analysed using HLSVD-PRO and peak areas were calculated. Voxels with CRLB/amplitude < 20% were selected and their metabolite peaks were quantified. The calculated CRLB of both methods for all the volunteers was provided in S2 Appendix. Means and standard deviations of NAA/Cr and Cho/Cr ratios of both CSI and RAPID-SI (*R* = 8) reported in Tables 2 and 3, and calculated from the ROI (Fig 7A) across all the volunteers were comparable. The left box plot in Fig 8 displays the mean NAA/Cr ratio inside the ROI for both CSI and RAPID-SI for all the volunteers. The CSI median value was 2.01, ranging between 1.9 and 2.17, whereas with RAPID-SI (*R* = 8), the median value was 1.99 varying between 1.92 and 2.15. No significant difference between both methods was found based on the p-value of 0.53 using the Wilcoxon signed-rank test. Similarly, the right box plot in Fig 8 displays the mean Cho/Cr ratio inside the ROI for both techniques across all the volunteers. The CSI median value was found to be 0.90, ranging between 0.84 and 0.97, whereas with RAPID-SI (*R* = 8), the median value was 0.89 varying between 0.82 and 0.95. No significant difference between the two methods was found based on the p-value of 0.06.

**Table 2:** Mean (± standard deviation) of the *in vivo* NAA/Cr ratios across all the volunteers for CSI and RAPID-SI (*R* = 8) inside the ROI.

NAA/Cr	Subject 1	Subject 2	Subject 3	Subject 4	Subject 5	Subject 6	Subject 7	Subject 8
**CSI**	**2.15 ± .08**	**1.90 ± .08**	**1.92 ± .15**	**2.03 ± .11**	**2.17 ± .11**	**2.03 ± .14**	**2.00 ± .15**	**1.91 ± .22**
**RAPID-SI (*R* = 8)**	**2.13 ± .06**	**1.92 ± .07**	**1.92 ± .17**	**2.00 ± .14**	**2.15 ± .13**	**2.05 ± .13**	**1.98 ± .14**	**1.93 ± .20**

**Table 3:** Mean (± standard deviation) of the *in vivo* Cho/Cr ratios across all the volunteers for CSI and RAPID-SI (*R* = 8) inside the ROI.

Cho/Cr	Subject 1	Subject 2	Subject 3	Subject 4	Subject 5	Subject 6	Subject 7	Subject 8
**CSI**	**0.92 ± .05**	**0.94 ± .03**	**0.97 ± .02**	**0.86 ± .04**	**0.94 ± .03**	**0.88 ± .14**	**0.86 ± .03**	**0.84 ± .06**
**RAPID-SI (*R* = 8)**	**0.90 ± .03**	**0.92 ± .02**	**0.95 ± .02**	**0.88 ± .04**	**0.92 ± .03**	**0.88 ± .13**	**0.84 ± .03**	**0.82 ± .07**

To further demonstrate the usefulness of the proposed method, a 3D (16x16x4) RAPID-SI and CSI dataset, collected from two volunteers using the same acquisition parameters as the 2D, except for TR = 1800 ms, and *N*_*avg*_ = 1. The resolution in the slice direction was reduced to 4, due to the long acquisition time required by CSI. S2 Fig displays spectra selected from one of the slices, with a zoom on the ventricle region showing the conformity of spectral content with the anatomy of both RAPID-SI and CSI. The acquisition time with the CSI was 30 min 43 sec, reduced to 3 min 50 sec with the RAPID-SI.

## Discussion

The proposed technique was compared to CSI in terms of acquisition time, spectral resolution and data sensitivity. Both RAPID-SI and CSI, use the same encoding principle (phase encoding), but differ in their time of application. The CSI performs the encoding prior to signal acquisition, whereas the RAPID-SI achieves the same task during data collection to segment the signal into sequential short time duration encoded sub-signals. This results in an SNR decrease by approximately the factor √R at the acquisition level (Eq 7), which is compensated during data pre-processing. The amount of noise in the output is minimal due to the use of noiseless full time pre-processed signals estimated from the corresponding acquired sub-signals along with the acquired reference signal at *k* = 0. The amplitudes are accurately estimated as demonstrated by simulation, phantom and *in vivo* results.

Other techniques like the Multi-Spin-Echo Proton Spectroscopic Imaging technique, EPSI, SPICE and others have been developed to accelerate conventional MRSI. However, in the Multi-Spin-Echo Proton Spectroscopic Imaging technique [4,5], spectral resolution is reduced because of the shortened readout and spatial blurring occurs due to signal modulation caused by *T*_2_ relaxation. This technique is most useful for metabolites having long *T*_2_ values such as NAA and Choline and the maximum acceleration factor is also typically only 2 to 3. In a conventional EPSI sequence [6], the acceleration factor is higher. For the example of a 2D 16x16 data, the acquisition time can be accelerated by a factor of 16 and even higher for higher resolution but at the penalty of reduced SNR by a factor determined by the dwell time of acquisition. It also requires high performance gradient systems with high slew rate. The SPICE technique [8] has been proposed to improve the spectral resolution and SNR as compared to EPSI with reduced acquisition time but still it requires the sophisticated gradient system for data acquisition along with sophisticated post processing method. RAPID-SI uses an acceleration factor of 8 for any spatial resolution and recovers the metabolite information. Higher acceleration factor reduces the performance of the technique, with no requirement of high performance gradients, used in other techniques such as EPSI.

The short time duration of the encoded sub-signals are expected to carry similar frequency and damping factors of these metabolites. The use of a reference signal *AS*_0_ (*k* = 0,*N*_*full*_ = 512) is therefore sufficient to accurately estimate the values of the frequency and damping factors, which are used in the data reconstruction process. It is to be noted that data analysis and quantification can only be performed once the pre-processing is performed, without which the data will be very low in SNR and with poor spectral resolution leading to inaccurate data analysis. Therefore, the pre-processing is a mandatory step and must follow data acquisition for accurate data quantification.

One should notice that because the reference signal is the sum of the signals from all the voxels at the centre of k-space, the estimated frequency and damping factor values for each metabolite is the average value across all the voxels. The B_0_ inhomogeneity may cause discrepancy of these values, which is reflected on the corresponding estimated average. However, this average value should remain constant across the encoded sub-signals, since each encoded sub-signal is also the sum of the sub-signals of all the voxels but at a given k-space value. This is comparable to CSI, where the collected signals are the sum of all the voxels at each encoding step with frequency and damping factor averaged. The well-known Lorentzian model has been chosen to represent the spectral lines. However, the method is flexible to incorporate any other signal model (e.g. Gaussian, Voigt), and therefore, any source of signal deformation (e.g. field inhomogeneity) could be included in the signal model to improve the method outcome. In our pre-processing software, we have chosen the HLSVD method, which is a reliable method and proven to provides accurate results. Other analysis procedures such as LCModel [26], or jMRUI [27], have to be adapted to be used as a substitute in the pre-processing step. These methods can also be used for data quantification following the pre-processing step.

The duration of the acquisition window was set to the average *T*_2_ values of the main brain metabolites (NAA, Cr, Cho, mI, and lac) obtained at 3 Tesla, which is approximately 250 ms. This is found to preserve peak information in all the collected sub-signals, especially those acquired at high k space values (edges of k-space), and therefore, provide accurate estimation of signal parameters using the pre-processing algorithm. As a consequence, the suitable *N*_*full*_ value was found to be 512 data points for a receiver bandwidth equal to 2 kHz, and the best acceleration factor was 8. Higher acceleration factor is possible (*R* = 16). However, this reduces the number of points in *N*_*sub*_ (inversely proportional to *R*), and degrades the data quality, by affecting the quantification results as shown in the simulation and *in vitro* results.

The encoding efficiency of the proposed technique is subject to the *T*_2_ values of the metabolites. Given that the k-space is filled during the acquisition window, the number of k-space data points used to spatially encode each metabolite signal is proportional to its corresponding *T*_2_ value. Signal components with long *T*_2_ are sampled by several data points in k-space, whereas those with short *T*_2_ will have less points to characterize them. This may cause a spatial blurring (voxel bleeding) of the metabolite maps with short *T*_2_. To overcome this problem, a deconvolution between a sinc-like function with width at half maximum (WHM) inversely proportional to the *T*_2_ values of the blurred metabolite signals and their corresponding maps was performed to recover the original spatial distribution of the metabolites.

Several k-space trajectories are possible (e.g. Cartesian, radial, and spiral) using this method. For the sake of simplicity, the Cartesian scheme was chosen, starting from one edge of the k space, which coincides with the origin of the decaying part of the spin echo and moving towards the other edge while recording the sub-signals. In this manner, a balance in term of signal intensity, and therefore an SNR level between the acquired sub-signals is maintained to allow for better estimation of the signal parameters when applying the pre-processing procedure. Other k-space paths can also be explored in future and compared in terms of acquisition time, data sensitivity and reconstruction.

The developed method was tested and validated by performing simulated, phantom and *in vivo* experiments. As noticed from simulation, the performance of method drops at very low SNR values (SNR = 4.5) and high level of field inhomogeneity (more than 8Hz). However, if these severe conditions are encountered, the acceleration factor is lowered. Compared to CSI, this method achieves a reduction in acquisition time, which is proportional to *R*, while providing accurate metabolite quantification results. Furthermore, the method is flexible to be combined with other acceleration methods, such as parallel imaging.

It will be more beneficial if a larger comparative study is conducted and other high speed techniques such as PEPSI, and SPICE methods are used, in order to compare their performance in terms of acquisition time, SNR, resolution, hardware requirements and domain of application with the RAPID-SI. In the future, we plan to extend this study for clinical applications along with comparison to other fast spectroscopic imaging techniques.

## Supporting information

S1 Fig Diagram showing the map of linewidth variations due to the effect of field inhomogeneity across all the voxels in the ROI. (A) difference in linewidth of the NAA, Cr, Cho peaks from two voxels inside the ROI, (B) voxel near the edge and (C) voxel near the centre.
(TIF)

S2 Fig A 3D 16x16x4 *in vivo* data (one slice shown). Selected spectra from the red boxes near the ventricle region are shown for the CSI (left) in red and RAPID-SI (*R* = 8) (right) in blue.
(TIF)

S1 Appendix Calculated SNR values *in vivo* with CSI and RAPID-SI (*R* = 8). (XLSX)

S2 Appendix Calculated CRLB values of NAA, Cr and Cho peak amplitude estimates *in vivo* with CSI and RAPID-SI (*R* = 8). (XLSX)
