# Supplementary figures and images for: Reduction of Acquisition time using Partition of the sIgnal Decay in Spectroscopic Imaging technique (RAPID-SI)
Source: PLoS One. 2018 Nov 7;13(11):e0207015. doi: 10.1371/journal.pone.0207015 (PMC6221315; doi:10.1371/journal.pone.0207015)

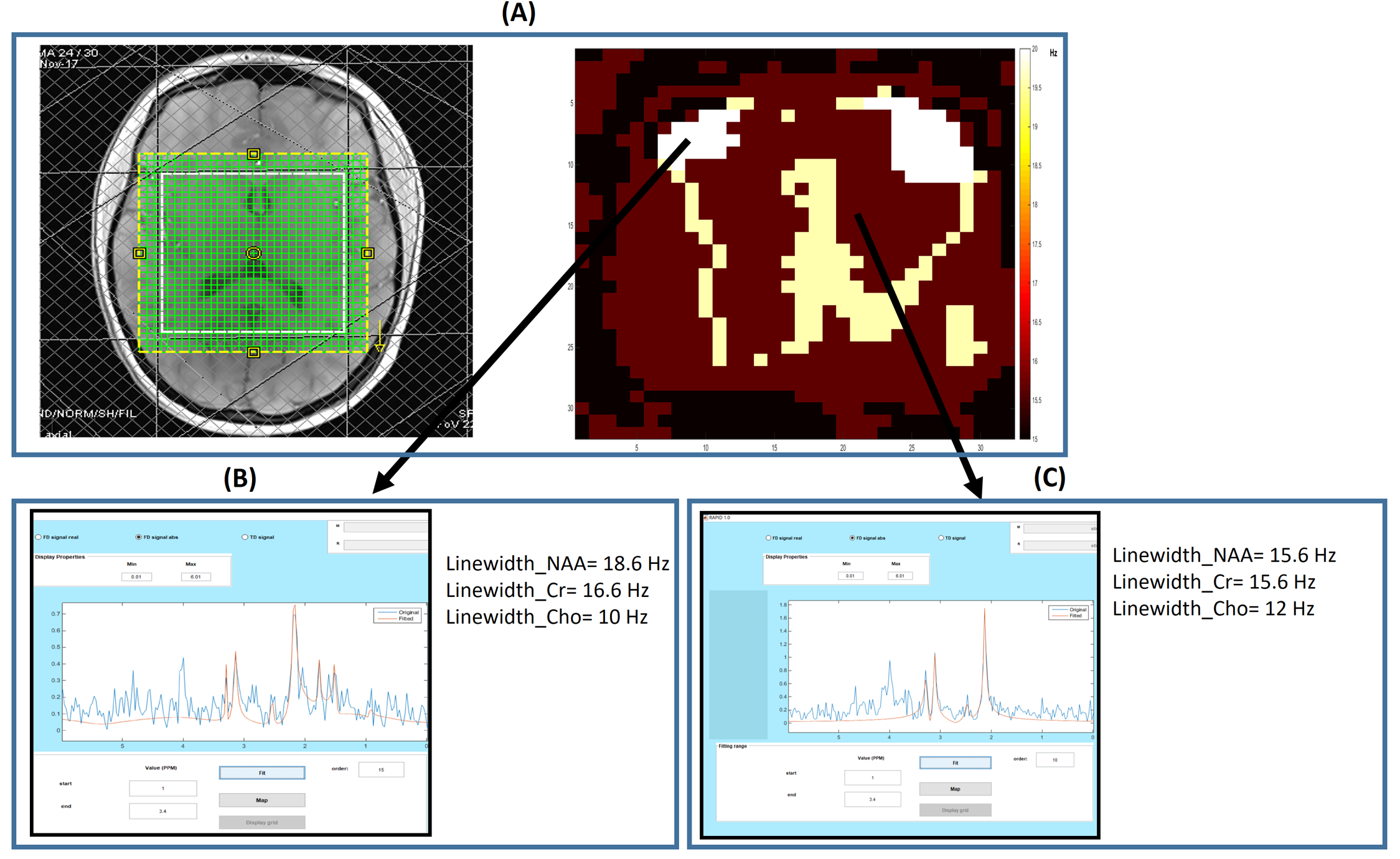

Supplement: S1 Fig — (A) difference in linewidth of the NAA, Cr, Cho peaks from two voxels inside the ROI, (B) voxel near the edge and (C) voxel near the centre. (TIF) [file pone.0207015.s001.tif]

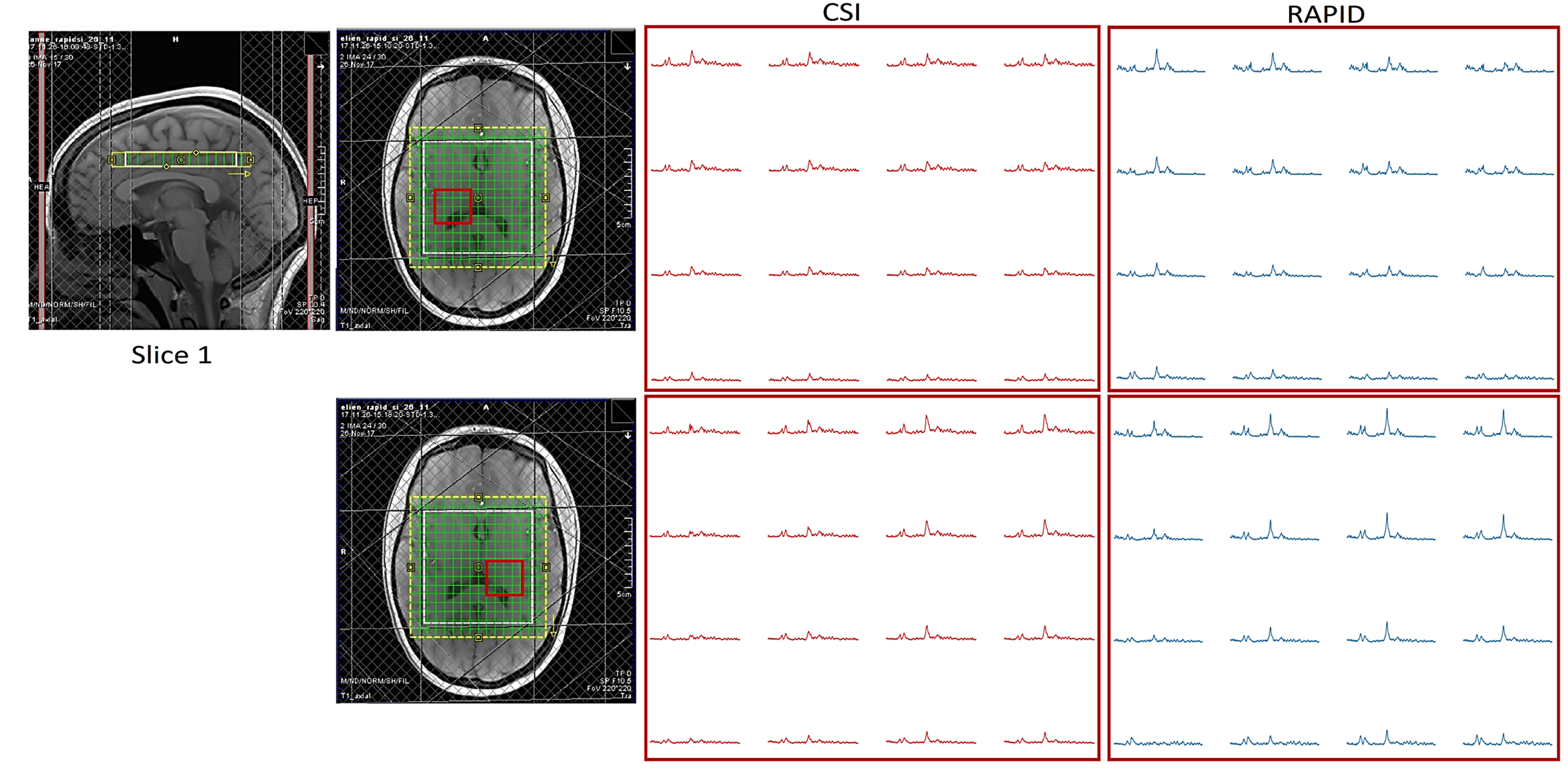

Supplement: S2 Fig — Selected spectra from the red boxes near the ventricle region are shown for the CSI (left) in red and RAPID-SI (R = 8) (right) in blue. (TIF) [file pone.0207015.s002.tif]
